# Host habitat rather than evolutionary history explains gut microbiome diversity in sympatric stickleback species

**DOI:** 10.3389/fmicb.2023.1232358

**Published:** 2023-10-12

**Authors:** Aruna M. Shankregowda, Prabhugouda Siriyappagouder, Marijn Kuizenga, Thijs M. P. Bal, Yousri Abdelhafiz, Christophe Eizaguirre, Jorge M. O. Fernandes, Viswanath Kiron, Joost A. M. Raeymaekers

**Affiliations:** ^1^Faculty of Biosciences and Aquaculture, Nord University, Bodø, Norway; ^2^School of Biological and Behavioural Sciences, Queen Mary University of London, London, United Kingdom

**Keywords:** host microbiota, sticklebacks, evolutionary history, host habitat, adaptation, symbiotic microbiota, adaptive divergence

## Abstract

Host-associated microbiota can influence host phenotypic variation, fitness and potential to adapt to local environmental conditions. In turn, both host evolutionary history and the abiotic and biotic environment can influence the diversity and composition of microbiota. Yet, to what extent environmental and host-specific factors drive microbial diversity remains largely unknown, limiting our understanding of host-microbiome interactions in natural populations. Here, we compared the intestinal microbiota between two phylogenetically related fishes, the three-spined stickleback (*Gasterosteus aculeatus*) and the nine-spined stickleback (*Pungitius pungitius*) in a common landscape. Using amplicon sequencing of the V3-V4 region of the bacterial 16S rRNA gene, we characterised the α and β diversity of the microbial communities in these two fish species from both brackish water and freshwater habitats. Across eight locations, α diversity was higher in the nine-spined stickleback, suggesting a broader niche use in this host species. Habitat was a strong determinant of β diversity in both host species, while host species only explained a small fraction of the variation in gut microbial composition. Strong habitat-specific effects overruled effects of geographic distance and historical freshwater colonisation, suggesting that the gut microbiome correlates primarily with local environmental conditions. Interestingly, the effect of habitat divergence on gut microbial communities was stronger in three-spined stickleback than in nine-spined stickleback, possibly mirroring the stronger level of adaptive divergence in this host species. Overall, our results show that microbial communities reflect habitat divergence rather than colonisation history or dispersal limitation of host species.

## 1. Introduction

Host-associated microbiota play a key role in the biology, ecology and evolution of their hosts (Alberdi et al., 2016; Henry et al., 2021). In vertebrates, gut microbial symbionts support diverse functions such as host immunity, organ development, digestion, and physiology (Sommer and Bäckhed, 2013). There is growing evidence that symbiont metagenomes help their hosts to adapt to new environmental conditions and expand their ecological niches, thus contributing to their broad ecological success (Jackson et al., 2022; Cornwallis et al., 2023). Thus, the responses of an organism to its environment are not only based on the interaction between the genotype and the environment (McFall-Ngai et al., 2013), but are also a function of its symbiotic microbiota (Bordenstein and Theis, 2015; Alberdi et al., 2016; Kolodny and Schulenburg, 2020).

In turn, microbiome composition is strongly influenced by both host characteristics as well as the environment (Sullam et al., 2012; Small et al., 2022). For instance, the composition of gut microbial communities may change with the age of the host (Lokesh et al., 2019), and is influenced by abiotic factors such as temperature and pollution (Claus et al., 2016; Sepulveda and Moeller, 2020), and by biotic factors including parasites and diet (Maslowski and Mackay, 2011; Leung et al., 2018; Hahn et al., 2022; Hodžić et al., 2023).

At the population level, both ecological and evolutionary processes such as selection, dispersal, and ecological drift shape the gut microbiome (Kohl, 2020). Previous studies mainly focused on how these various factors affect the microbiome, but did not explicitly investigate the relationship between host and microbiota in natural populations with known evolutionary history, and population characteristics under different environmental conditions. This limits our understanding of the larger evolutionary patterns that occur between hosts and their associated microbiota in natural populations. Furthermore, most studies focus on a single host species, and therefore cannot simultaneously characterise host-specific and environmental effects on microbiota.

Stickleback fishes (Gasterosteidae) are a group of small fishes that are found in both marine and freshwater habitats (Gibson, 2005). The three-spined stickleback (*Gasterosteus aculeatus* Linnaeus, 1758) and the nine-spined stickleback (*Pungitius pungitius* Linnaeus, 1758) are important model organisms for the study of natural selection and adaptive evolution (Gibson, 2005; Raeymaekers et al., 2005; DeFaveri et al., 2012; Feulner et al., 2013; Merilä, 2013; Fang et al., 2021). Both species diverged around 26 million years ago (Varadharajan et al., 2019), but have overlapping habitat requirements (Zander, 1990), diet preferences (Hart, 2003) and parasite communities (Raeymaekers et al., 2008; Thorburn et al., 2022). Studies in three-spined stickleback have identified several environmental and host-specific factors that correlate with the diversity and community structure of the gut microbiota. In a North American three-spined stickleback population, gut microbiota composition was associated with sex, diet, ecotype, and habitat (Bolnick et al., 2014b,c; Smith et al., 2015). In addition, there was a relationship between gut microbiota and the level of polymorphism at the major histocompatibility genes that play a key role at the onset of adaptive immune response (Bolnick et al., 2014a). Furthermore, across three Canadian benthic-limnetic stickleback pairs, microbial communities cluster more by ecotype than by lake, suggesting that host–microbe interactions play a potential role in host adaptation (Rennison et al., 2019). No studies have been performed thus far on the gut microbiome of the nine-spined stickleback.

Three-spined and nine-spined stickleback populations in Belgium and Netherlands co-occur in a wide range of habitats, including estuaries, creeks, rivers, ditches, and ponds (Raeymaekers et al., 2017; Bal et al., 2021; Thorburn et al., 2022). In this part of their distribution range, both species differ markedly in the strength and nature of local adaptation, where brackish water and freshwater populations show stronger morphological and genomic differentiation in the three-spined stickleback than in the nine-spined stickleback (Raeymaekers et al., 2017; Bal et al., 2021). This implies that the three-spined stickleback might be more sensitive to natural selection, and entails the possibility that the nine-spined stickleback relies more on non-genetic mechanisms for coping with varying environmental conditions. For instance, it could be that microbiome-mediated plasticity facilitates the freshwater-brackish water transition in nine-spined stickleback.

In this study, we investigate to what extent the gut microbiome of natural populations reflects these different evolutionary histories and putative underlying adaptive contexts of the two host species. To do so, we compare their microbial communities within and between the two main habitat types where they co-occur. Specifically, the study of the diversity of the gut microbial community (alpha diversity) at locations of sympatric host species, as well as the level of divergence in community composition (beta diversity) between freshwater and brackish water populations, allows us to test to what extent intestinal microbial communities are shaped by host characteristics, environmental factors, and their interaction. We hypothesised that if the composition of the gut microbiome is mostly driven by environmental characteristics, there should be substantial overlap in microbial composition between the two host species. Alternatively, if the composition of the gut microbiome mostly reflects the host’s evolutionary history, we expect microbial compositions unique to each host species, with differentiation patterns that mirror population genomic differentiation. We thus characterised the shared and unique microbiota of the two stickleback species, and tested for species-specific and habitat-specific effects on the composition of the microbiota of the two host species.

## 2. Materials and methods

### 2.1. Sample collection

**Figure 1:**
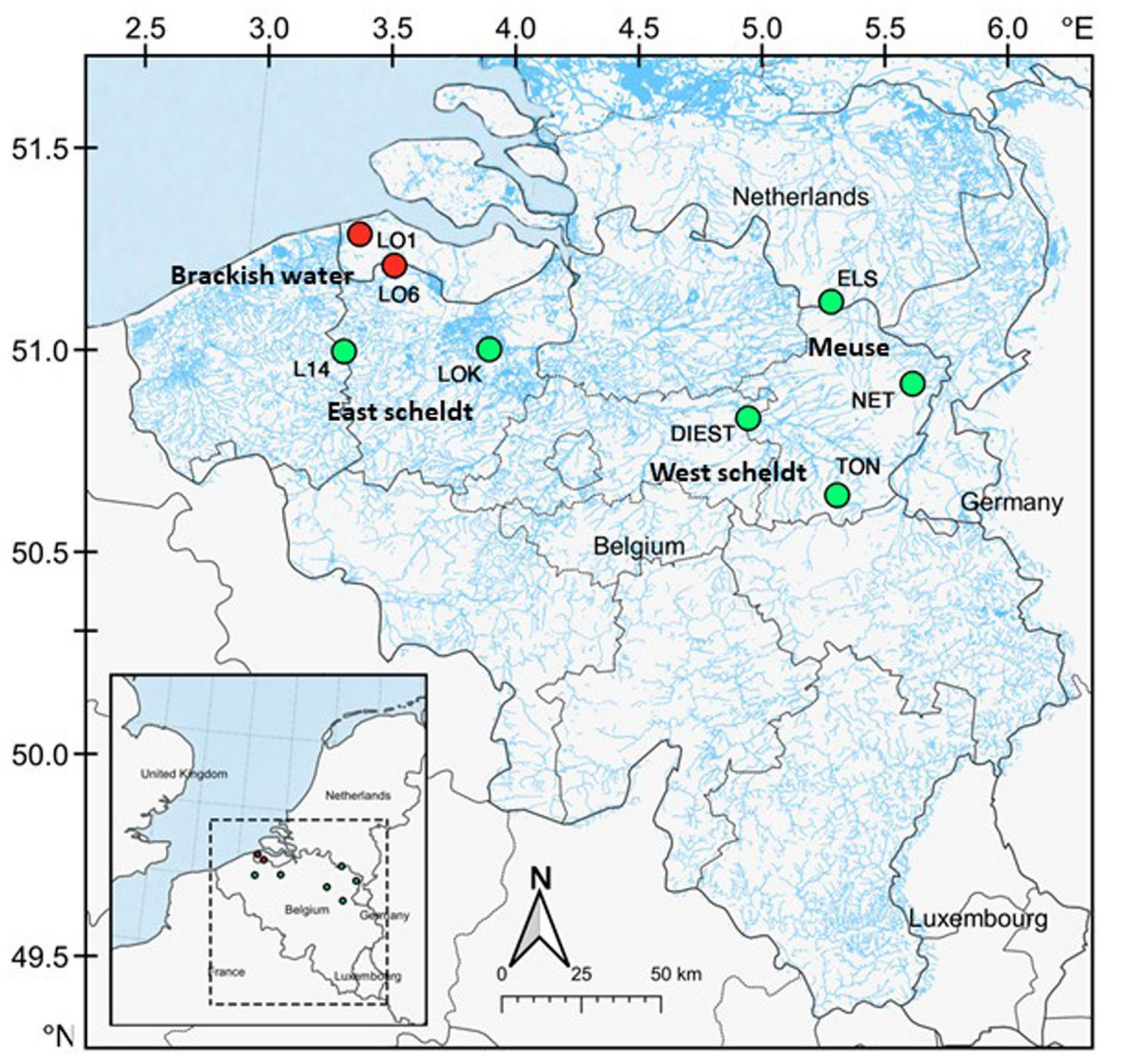
Map of the study area. A total of eight locations were sampled across Belgium and Netherlands. Locations in red are brackish water (conductivity ⩾1,000 μS cm^−1^), and locations in green are freshwater.

The study system is located in brackish and freshwater habitats of Belgium and Netherlands, including estuaries, creeks, rivers and ditches, where both three-spined stickleback and nine-spined stickleback co-exist. All the samples were collected across eight locations, including two brackish water and six freshwater locations (Figure 1; Supplementary Table S1). The two brackish water locations are located in the Belgian–Dutch coastal lowlands (LO1 and LO6). The freshwater locations were selected from the Meuse basin (ELS and NET), the Eastern Scheldt basin (DIEST and TON), and the Western Scheldt basin (L14 and LOK). Fish from these locations were sampled in the autumn of 2020. From each location, 16 individuals per species (256 individuals in total) were collected using a dip net. Sticklebacks were flash-frozen in dry ice after being killed with a lethal dose of buffered Tricaine methanesulfonate (MS-222, Syndel, United States) with procedural approval from the Ethical Commission Animal Experiments of KU Leuven Belgium. The samples were transferred to Nord University (Bodø, Norway) in dry ice. The fish were thawed on ice, and they were dissected to collect the posterior intestine. In contrast to the anterior intestine, the posterior intestine appears to have a more stable core microbial community during unperturbed conditions. Because of its stability, it is a good option for comparative studies across various populations and environments (Ray and Ringø, 2014). Any visible gut content in the posterior intestine was removed and then intestine samples were transferred to cryotubes using sterile instruments. The samples were stored at –80°C until further use.

### 2.2. DNA extraction and sequencing

Genomic DNA was extracted from individual posterior intestinal tissues using DNeasy Blood & Tissue Kit (Qiagen, Hilden, Germany) as per the manufacturer’s instructions with slight modifications. The whole posterior intestinal tissue was incubated overnight (56°C) to allow the tissue lysis. The tissue lysate was eluted with 25 μL pre-heated (50°C) elution buffer for 5 min before centrifugation to enhance the DNA yield. Then, the purity and concentration of extracted DNA was determined using NanoDrop OneC Microvolume UV–Vis Spectrophotometer (Thermo Fisher Scientific – Invitrogen, Waltham, MA, United States) and Qubit^®^ dsDNA HS assay kit (Thermo Fisher Scientific), respectively.

The resulting DNA was amplified using the specific bacterial primers 341F (5’CCTACGGGNGGCWGCAG 3′) and 805R (5’GACTACNVGGGTWTCTAATCC 3′) flanked by overhang Illumina adapters targeting the hypervariable V3–V4 region (~460 bp). All PCR reactions were performed with 25 μL reaction volume consisting of 12.5 μL AmpliTaq gold 360 Master Mix (Thermo Fisher Scientific), 1 μL (10 μM) of each barcoded PCR primer pair and 2–20 ng of DNA template (Siriyappagouder et al., 2018). In case of the negative control, 2 μL PCR grade H_2_O was used instead of a DNA template. The PCR products were visualised on 1.5% (w/v) agarose gel, and positive bands (~550 bp) were excised from the gel and purified using the QIAquick Gel Extraction Kit (Qiagen) according to the manufacturer’s instructions.

The first PCR product was used as template for a second PCR (8 cycles, 16S Metagenomic Sequencing Library Preparation, Illumina). This step was done to add dual indices and Illumina sequencing adapters (Nextera XT Index Primers, Illumina, San Diego, California, United States). Amplified PCR products were purified using Mag-Bind TotalPure NGS (Omega Bio-tek, United States) to obtain the amplicon libraries (Sample/Beads ratio – 1/1.12). In the last step, all amplicon libraries were pooled in equimolar concentrations. Fragment size distribution, quality and quantity of pooled library were assessed using the TapeStation 2200 (Agilent Technologies, Santa Clara, CA, United States). Furthermore, the pooled libraries were quantified using the KAPA Library quantification kit (Roche, Basel, Switzerland) and the Qubit^®^ dsDNA HS assay kit (Thermo Fisher Scientific). Finally, 300 bp paired-end sequencing was performed at the Norwegian Sequencing Centre on an Illumina MiSeq platform using the MiSeq^®^ reagent kit (Illumina).

### 2.3. Bioinformatic analyses

The sequenced paired-end reads were processed with the Quantitative Insights Into Microbial Ecology 2 (QIIME 2–2022.8) tool (Bolyen et al., 2018, 2019). Paired-end sequences were imported, quality controlled and merged (see Supplementary Table S1) using the DADA2 algorithm in QIIME 2 (−-p-trim-left-f 13 --p-trim-left-r 13 --p-trunc-len-f 240 --p-trunc-len-r 240) (Callahan et al., 2016). The silva database (version 138) (Quast et al., 2012) trained with a naive Bayes machine-learning classifier (Robeson et al., 2021) was used to assign the taxonomy in QIIME 2. The generated Amplicon Sequence Variants (ASVs) table, taxonomy table, and phylogenetic tree were imported and merged into a phyloseq dataset object in R using qiime2R scripts (Bisanz, 2018) for further analysis. The resulting ASVs were subsequently filtered by removing singletons, unassigned ASVs, and ASVs assigned to Archaea, Euryarchaeota, or chloroplast DNA. Subsequently, only samples containing at least 5 distinct ASVs per sample were retained for further analyses.

### 2.4. Data analyses

All statistical analyses were carried out in the R v4.2.1 language in the Rstudio environment v2022.12.0 + 353 (R Core Team, 2021; RStudio Team, 2021). The data analysis was performed using the functions from the packages *phyloseq* (McMurdie and Holmes, 2013) and *vegan* (Oksanen et al., 2013). Data visualisation was done using *ggplot2* (Wickham, 2016) and *microViz* (Barnett et al., 2021). In order to take into account the read variation across samples and prepare the data for further analysis, reads were rarefied to 9,000 reads per sample, except for the analysis of differential abundance of ASVs.

Our analyses aimed at (1) identifying the shared and unique microbial communities of the two species at the eight locations, and (2) testing for species-specific and habitat-specific effects on the composition of the microbiota in each host species. To do so, we first examined the overall taxon diversity, and then compared alpha and beta diversity across host species, habitats and locations.

#### 2.4.1. Taxon composition

For an initial understanding of the composition of the microbial communities, a Venn diagram was constructed to visualise the percentage of shared and unique ASVs across host species and habitats. To identify key differences between host species and locations, we determined the top five phyla in the entire dataset, and assessed which of the commonly reported genera in other fish microbiome studies were present. For both the top phyla and these selected genera, two-way ANOVA was used to test for differences in abundance between host species and locations. In addition, the *microbiome* package in R was used to calculate the core microbiota of each host species across eight locations. Here, core microbiota were defined as genera with a prevalence of at least 80%, and a detection level (relative abundance) of 0.10. Core genera were identified after comparing the core microbiota of each host species separately, across eight locations. Finally, to describe the difference in microbiome composition between species in each habitat, ASVs were pooled by freshwater and brackish water locations, for each host species separately. A Wald test implemented in the *DEseq2* package (Love et al., 2014) was then used to determine differential abundance based on non-rarified abundance data.

#### 2.4.2. Alpha diversity

Differences in alpha diversity of gut microbial communities between host species and sampling locations were calculated using three ecological diversity measures: Simpson diversity (dominant species), Chao1 diversity (species richness) and Shannon diversity (evenness of the community). Two-way ANOVA was used to test for the effect of species, location and the species × location interaction term on infracommunity alpha diversity. Finally, Pearson correlations were calculated to assess the association of alpha diversity with salinity and distance to the coast (km), and to test if alpha diversity in three-spined stickleback is correlated with alpha diversity in nine-spined stickleback.

#### 2.4.3. Beta diversity

β-diversity was estimated using weighted and unweighted UniFrac (phylogenetic) dissimilarity matrices (Lozupone and Knight, 2005; Lozupone et al., 2011). The use of unweighted UniFrac matrices increases the effect of rare ASVs by considering their presence or absence, while weighted UniFrac matrices take into consideration the abundance of the ASVs and, thus, can be strongly impacted by highly abundant ASVs, particularly if the bacterial phylogeny is separated by long branches (Lozupone and Knight, 2005; Lozupone et al., 2011). For comparison, we also added Bray–Curtis dissimilarity (non-phylogenetic) matrices. The differences between the bacterial communities in host species and populations were further visualised and compared with non-metric multidimensional scaling (NMDS) analysis. Using the same dissimilarity matrices, we then performed permutational multivariate ANOVAs using the adonis2 function in *vegan* R package to quantify the effects of species, location, and the species × location interaction term on the gut microbiota composition. Permutational multivariate ANOVAs were also conducted on each host species separately, this time to assess the effects of habitat and location (nested in habitat).

Furthermore, we tested for different spatial scenarios of microbiome differentiation, measured as Bray-Curtis distances (beta diversity). In scenario 1, we assessed whether more distant host populations harbour more dissimilar microbiome communities. This scenario was tested by correlating gut microbiome differentiation (Bray–Curtis dissimilarity matrix) with Euclidean distances among sampling locations. In scenario 2, we assessed whether host populations from different habitats (freshwater or brackish water) harbour more dissimilar microbiome communities. This scenario was tested by correlating gut microbiome differentiation with a theoretical matrix assigning value 0 to habitat similarity and value 1 to habitat dissimilarity. In scenario 3, we tested whether host populations with a different freshwater colonisation history harbour more dissimilar microbiome communities, assuming that brackish water populations are ancestral and freshwater populations are derived [see raceme scenario in Raeymaekers et al. (2005)]. This scenario was tested by correlating gut microbiome differentiation with another theoretical matrix, assigning values 0 to brackish water population pairs (no freshwater colonisation history), values 0 to freshwater population pairs from the same watershed (same colonisation history), values 1 to brackish water-freshwater pairs (direct ancestry), and values 2 to freshwater populations from different watersheds (independent colonisation). The three scenarios were tested using Mantel tests. In addition, partial Mantel tests were used to test scenario 2 and 3 after accounting for scenario 1, i.e., the correlations between microbiome differentiation and the theoretical matrices of scenario 2 or scenario 3 were corrected for Euclidean distance.

## 3. Results

### 3.1. Taxon composition

A total of 5,987,681 high-quality reads were obtained from 253 samples with an average of 23,666 reads per sample (see Supplementary Table S1 and Supplementary methods).

**Figure 2:**
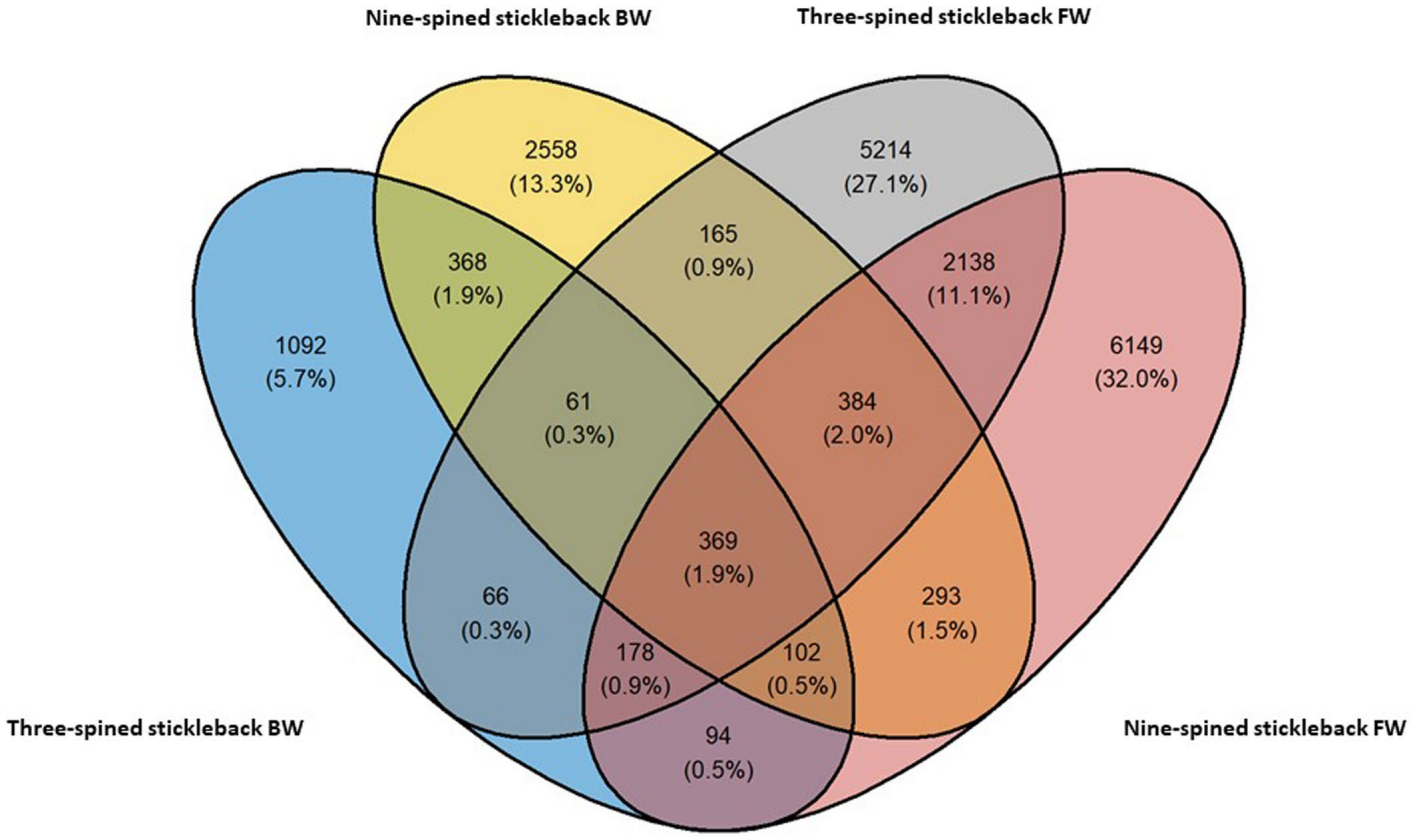
Shared and distinct ASVs of the three-spined and nine-spined stickleback populations from freshwater (FW) and brackish water (BW) habitat.

We observed a mix of shared and unique ASVs across host species and habitats. Nine-spined stickleback populations harboured 6,149 and 2,558 ASVs unique to freshwater and brackish water habitats, respectively (Figure 2). Three-spined stickleback populations harboured 5,214 and 1,092 ASVs unique to each habitat. Interestingly, the nine-spined stickleback shared more ASVs (5.9%) among both habitats than the three-spined stickleback (3.4%) (Figure 2). We found that populations of the two host species at the same locations share 7% to 21% ASVs (Supplementary Figure S1). The proportion of shared ranged from 11 to 19% in freshwater populations, and was both highest (LO1–21%) and lowest (LO6–7%) in brackish water populations (Supplementary Figure S1).

**Figure 3:**
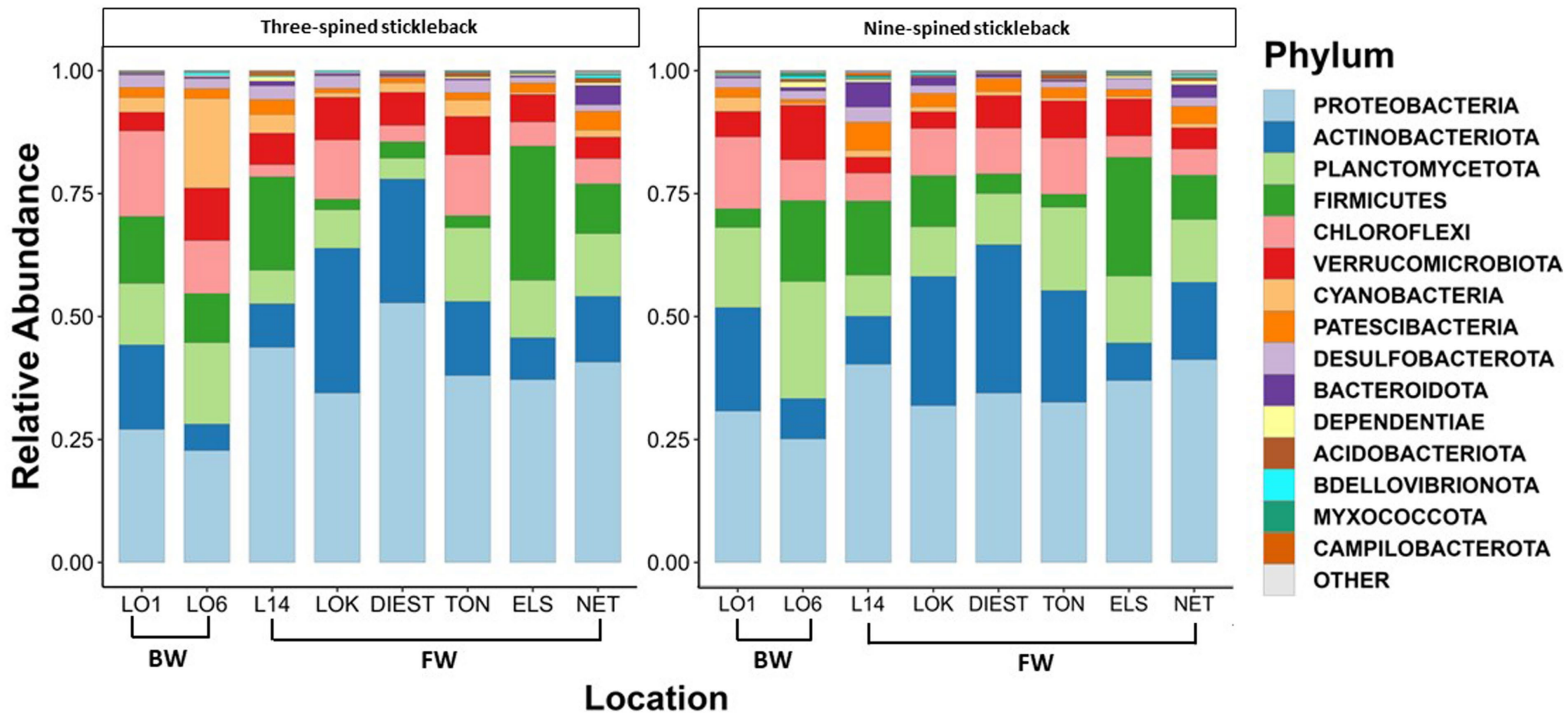
Relative abundance of the 15 most represented bacterial phyla in three-spined and nine-spined stickleback populations from brackish water (BW) and freshwater (FW) habitat.

**Figure 4:**
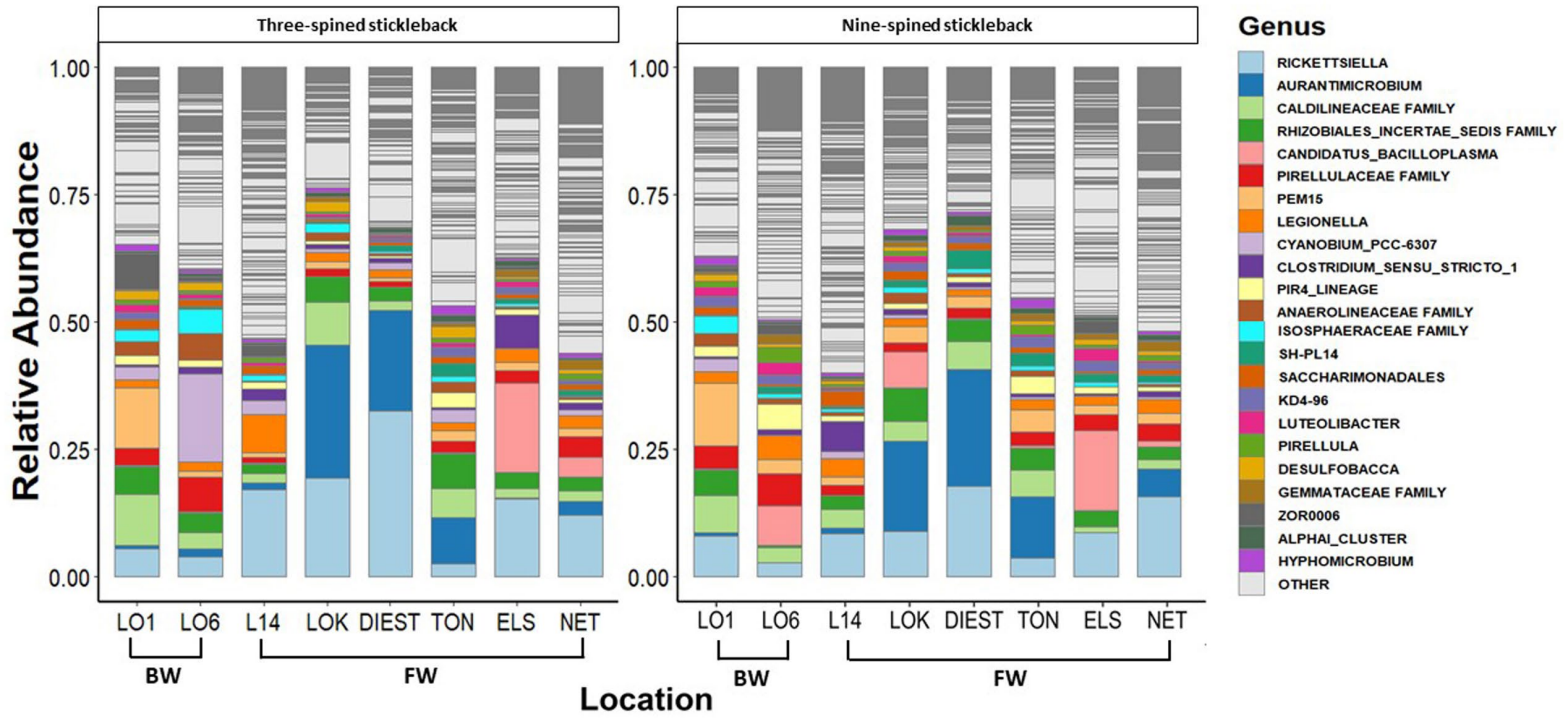
Relative abundance of the 23 most represented bacterial genera in three-spined and nine-spined stickleback populations from brackish water (BW) and freshwater (FW) habitat.

A total of 46 phyla were detected in both species, out of which 38 phyla were shared between two species. Eight phyla were unique to nine-spined stickleback, and two were unique to three-spined stickleback. We then identified the 15 most dominant microbial phyla and the 23 most dominant genera across host species and locations based on relative abundances (Figures 3, 4). Five phyla accounted for 80 to 90% of the community composition, irrespective of their host species. The most abundant phyla in the gut microbiota across the two host species and all locations were Proteobacteria, Actinobacteriota, Planctomycetota, Firmicutes, Chloroflexi and Verrucomicrobiota (Figure 3). The most abundant genera were *Rickettsiella, Aurantimicrobium, Candidatus bacilloplasma* and *PeM15* (Figure 4). Proteobacteria was the most abundant phylum in both brackish water and freshwater populations (Figure 3). The gut microbiota was highly location-specific, and was dominated by the *Rickettsiella* genus in both species.

Two-way ANOVA revealed variable effects of host species and location on the most abundant phyla and genera (Supplementary Tables S2, S3). For six out of ten taxa (five phyla and five genera), there was a significant location × host species interaction effect, with higher abundances for some locations in the three-spined stickleback, and higher abundances for other locations in the nine-spined stickleback (Supplementary Tables S2, S3). For the remaining taxa, there was a main effect of location, but there was no systematic difference in abundance between the host species.

### 3.2. Alpha diversity

**Figure 5:**
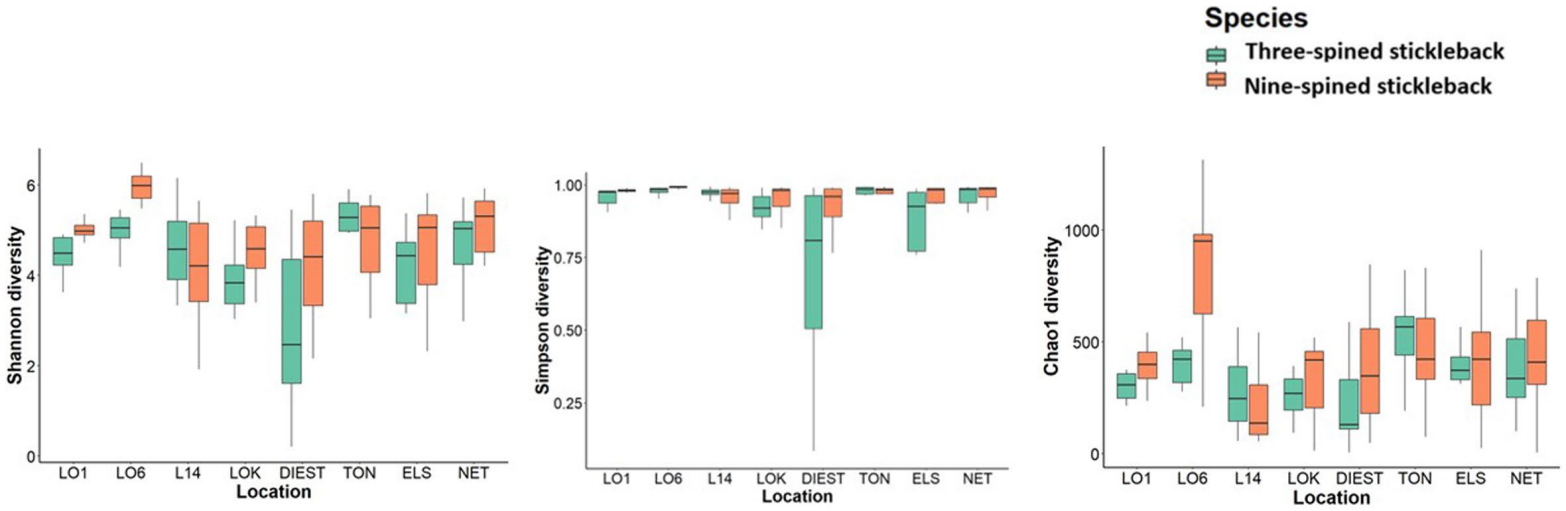
Alpha diversity of the bacterial communities in three-spined stickleback and nine-spined stickleback populations. The boxplots show minimum, lower quartile, median, upper quartile, and maximum values.

Overall, alpha diversity varied between host species and locations, and was on average higher in nine-spined stickleback (Table 1; Figure 5). There were significant differences in both Shannon and Simpson diversity between locations (Shannon diversity: *F*_7,198_ = 4.35, *p*-value = 0.0001; Simpson diversity: *F*_7,198_ = 3.56, *p*-value = 0.001, Table 1) and host species (Shannon diversity: *F*_1,198_ = 5.79, *p*-value = 0.016; Simpson diversity: *F*_1,198_ = 5.94, *p*-value = 0.015, Table 1). In case of Chao1 diversity, we observed significant differences among locations (*F*_7,198_ = 2.77, *p*-value = 0.008), and a significant location × host species interaction effect (*F*_7,198_ = 4.44, *p*-value = 0.0001, Table 1). Interestingly, while there were no significant correlations between alpha diversity and salinity or distance to coast in either host species, Simpson diversity in nine-spined stickleback was positively correlated with Simpson diversity in three-spined stickleback (Pearson correlation: *r* = 0.74, *p*-value = 0.03).

**Table 1:** Two-way ANOVA for the effect of location, host-species and the interaction between location, and host-species on alpha-diversity.

	Shannon diversity				Simpson diversity				Chao1 diversity			
	Df	Sum Sq	*F*	*p*-value	Df	Sum Sq	*F*	*p*-value	Df	Sum Sq	*F*	*p*-value
Location	7	44.48	4.35	**0.0001**	7	0.79	3.56	**0.001**	7	776,505	2.77	**0.008**
Host species	1	8.46	5.79	**0.016**	1	0.18	5.94	**0.015**	1	154,443	3.86	0.0505
Location: host species	7	12.28	1.20	0.30	7	0.22	0.99	0.43	7	1,240,889	4.44	**0.0001**
Residuals	198	288.9			198	6.28			198	7,904,347		

Df denotes degrees of freedom, *F* denotes *F* statistic, and Sum Sq denotes the variation attributed to the error. Significant results are shown in bold.

**Figure 6:**
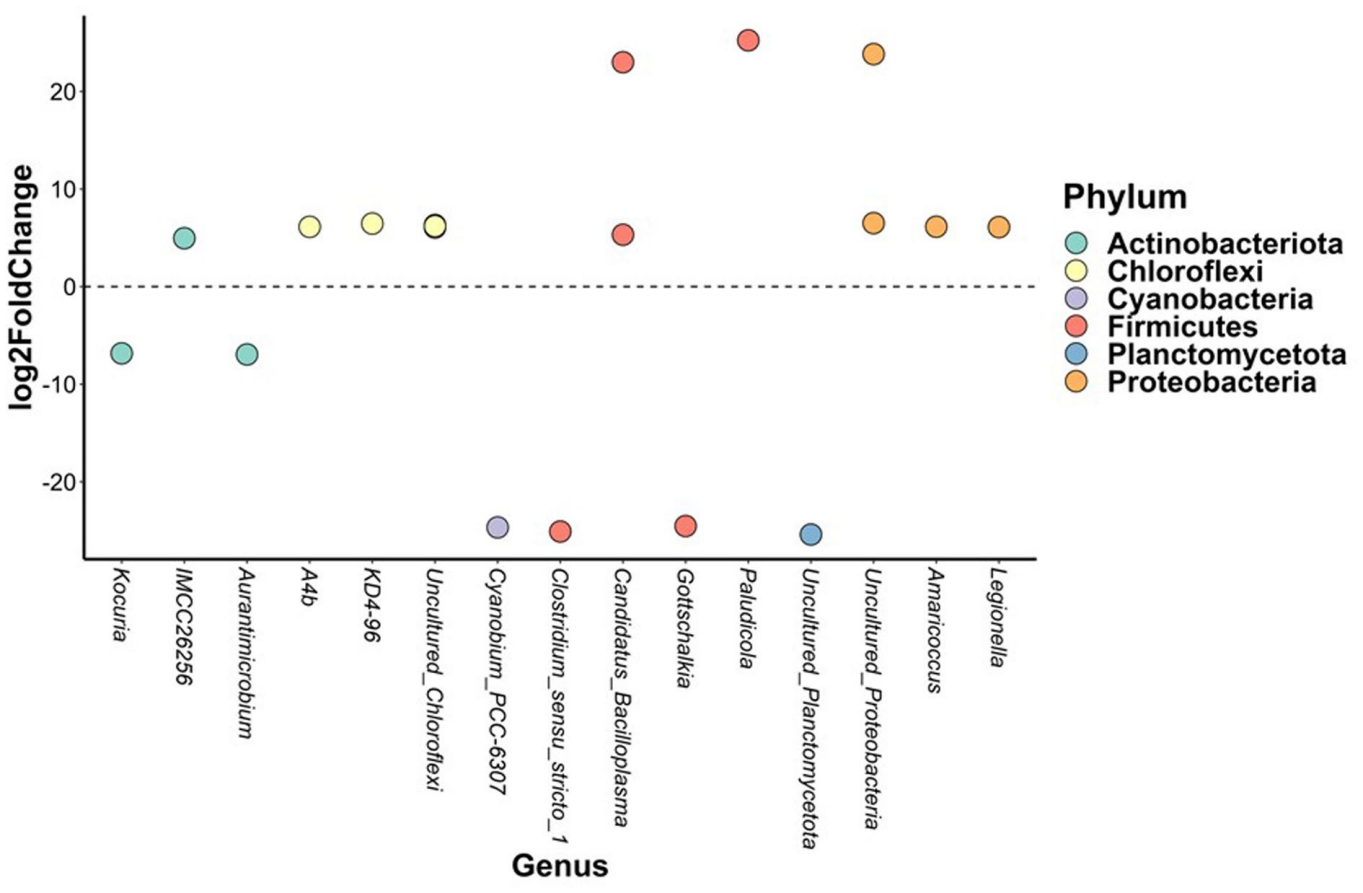
Differentially abundant amplicon sequence variants between brackish water populations from the three-spined stickleback and brackish water populations from the nine-spined stickleback. The X-axis labels are genus-level annotations of the microbes identified in the nine-spined stickleback.

In brackish water, the abundance of 19 bacterial ASVs from six phyla differed significantly between three-spined and nine-spined stickleback (Figure 6). Thirteen of those ASVs belonged to phyla Proteobacteria (*Legionella* and *Amaricoccus*), Actinobacteriota (*IMCC26256*), Firmicutes (*Candidatus_Bacilloplasma* and *Paludicola*), and Chloroflexi (*A4b* and *KD4-96*), and were more abundant in nine-spined stickleback (Figure 6). The other 6 ASVs were more abundant in three-spined stickleback, and belonged to the phyla Actinobacteriota (*Aurantimicrobium* and *Kocuria*), Firmicutes (*Clostridium_sensu_stricto_1* and *Gottschalkia*) and Cyanobacteria (*Cyanobium_PCC-6307*). In contrast, within the freshwater habitat, there were no differences in abundance between the two host species for any ASVs.

### 3.3. Beta diversity

**Figure 7:**
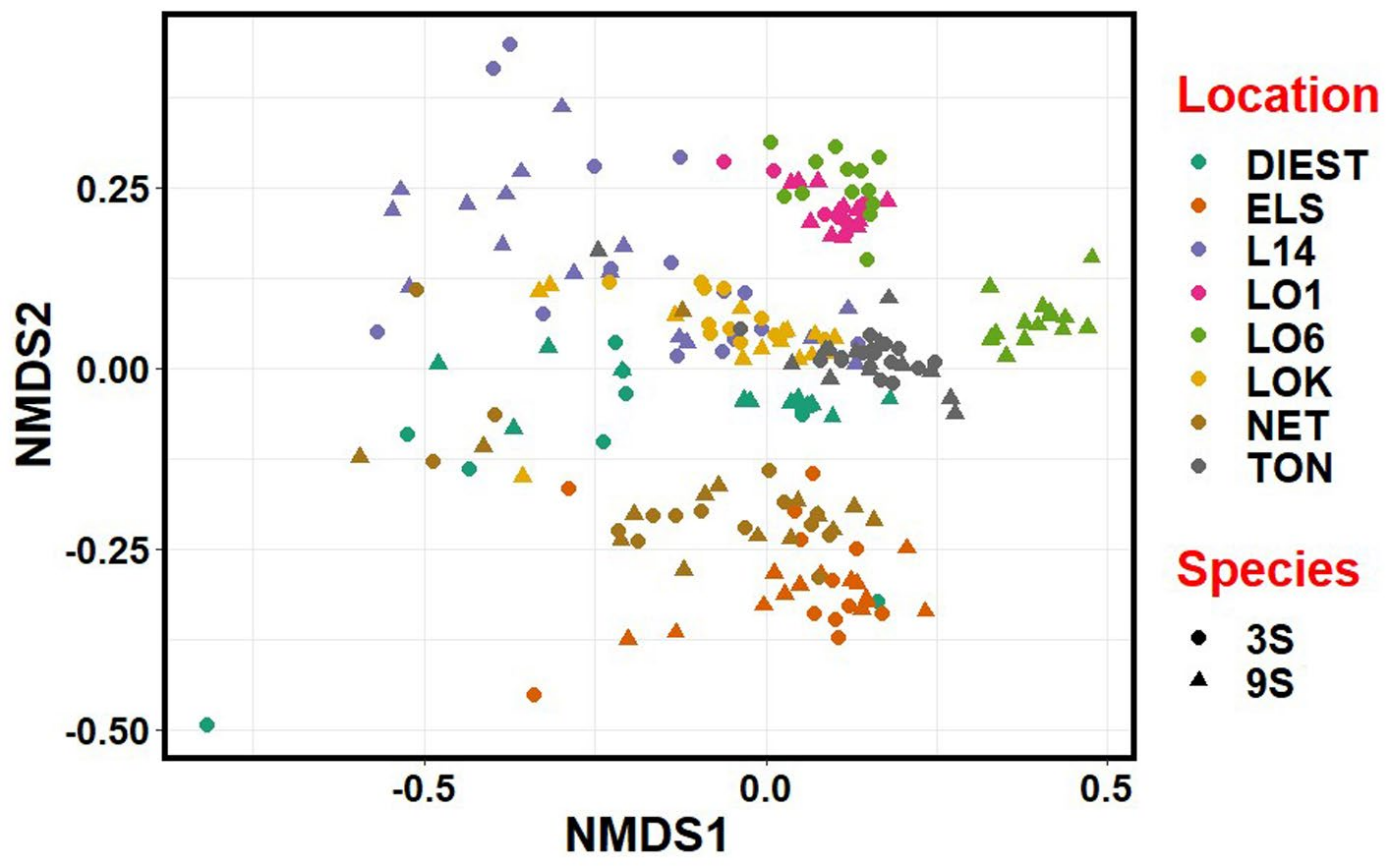
Non-metric multidimensional scaling (NMDS) plot based on Bray-Curtis distances between the microbiota communities from three-spined (3S) and nine-spined (9S) stickleback populations. Individual sticklebacks are labelled by location and species.

NMDS revealed that the microbial communities clustered more by location, river basin and habitat than by species (Figure 7). Accordingly, there was considerable overlap between the two host species from each location, except for one brackish water population (LO6) of the nine-spined stickleback where the microbial community was clearly distinct from other brackish water populations (Figure 7).

PERMANOVA revealed that location explained most of the variation in microbial communities (weighted UniFrac; *R*^2^ = 0.22, unweighted UniFrac; *R*^2^ = 0.156, Bray-Curtis; *R*^2^ = 0.22, *p*-value = ≤ 0.0001), followed by species (weighted UniFrac; *R*^2^ = 0.015, unweighted UniFrac; *R*^2^ = 0.009, Bray-Curtis; *R*^2^ = 0.009, *p*-value = ≤ 0.0001) and the species × location interaction term (weighted UniFrac; *R*^2^ = 0.057, unweighted UniFrac; *R*^2^ = 0.046, Bray-Curtis; *R*^2^ = 0.051, *p*-value = ≤ 0.0001) (Table 2; Supplementary Figure S2). PERMANOVA in each species separately indicated that habitat and location explained more variation in gut microbial composition in three-spined stickleback than in nine-spined stickleback for weighted UniFrac and Bray–Curtis dissimilarity matrices, but we observed no such difference for unweighted UniFrac matrices (Table 2; Supplementary Figure S2).

**Table 2:** PERMANOVA on distances (Weighted UniFrac, Unweighted UniFrac and Bray-Curtis) between microbial communities of individual three-spined and nine-spined sticklebacks from eight locations.

	Weighted UniFrac				Unweighted UniFrac				Bray-Curtis			
	Df	*F*	*R* ^2^	*p*-value	Df	*F*	*R* ^2^	*p*-value	Df	*F*	*R* ^2^	*p*-value
Location	7	9.08	0.225	**0.0001**	7	5.62	0.156	**0.0001**	7	8.75	0.22	**<0.0001**
Host species	1	4.36	0.015	**0.0001**	1	2.41	0.009	**0.0002**	1	2.63	0.009	**<0.0001**
Location: Host species	7	2.32	0.057	**0.0001**	7	1.66	0.046	**0.0001**	7	2.05	0.051	**<0.0001**
Residuals	198				198				198			
**Three-spined stickleback**												
Habitat	1	12.02	0.089	**0.0001**	1	4.18	0.035	**0.0001**	1	9.56	0.074	**<0.0001**
Location	6	5.06	0.226	**0.0001**	6	3.37	0.173	**0.0001**	6	4.60	0.21	**<0.0001**
Residuals	92				92				92			
**Nine-spined stickleback**												
Habitat	1	9.66	0.067	**0.0001**	1	5.83	0.043	**0.0001**	1	8.98	0.062	**<0.0001**
Location	6	4.63	0.193	**0.0001**	6	3.55	0.160	**0.0001**	6	4.91	0.20	**<0.0001**
Residuals	106				106				106			

Df denotes degrees of freedom and *F* denotes *F* statistic. Significant results are shown in bold.

**Figure 8:**
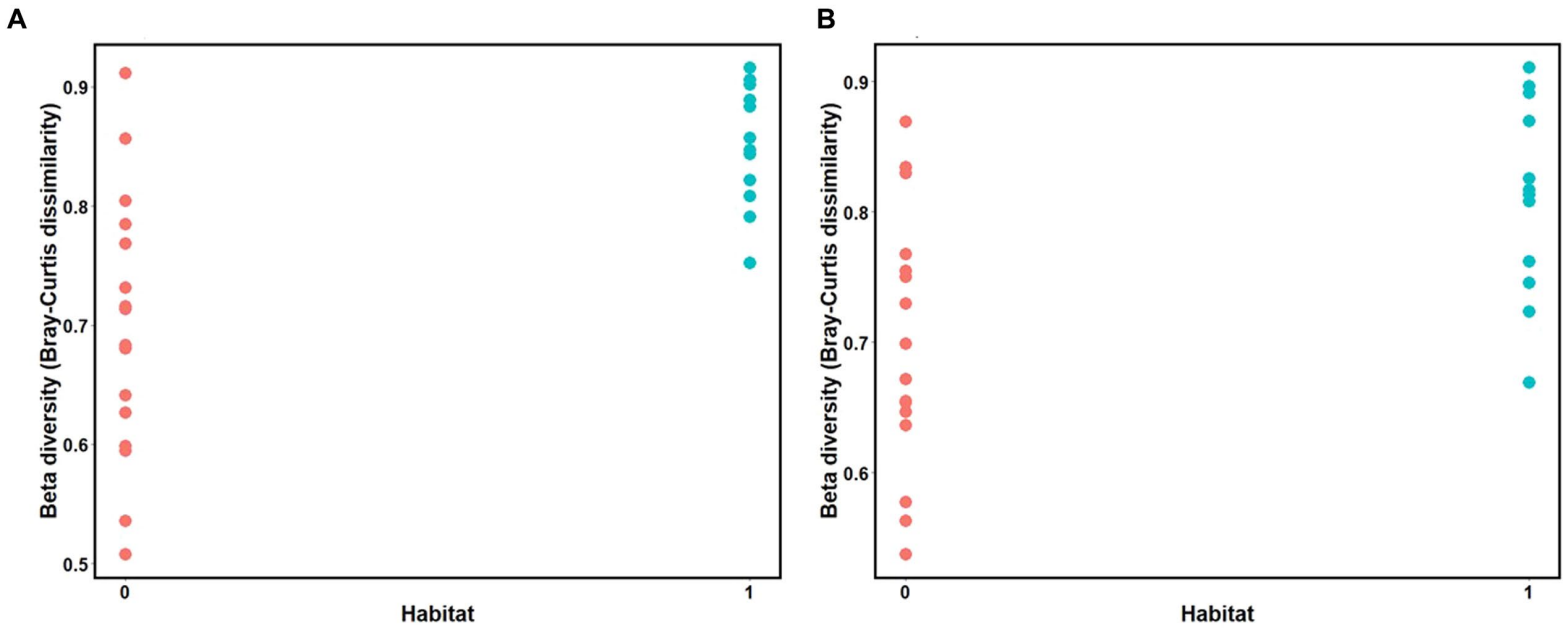
Mantel test for isolation by distance between the matrix of habitat dissimilarities and Bray Curtis dissimilarities. **(A)** Three-spined stickleback (*R* = 0.65, *p*-value = 0.03). **(B)** Nine-spined stickleback (*R* = 0.54, *p*-value = 0.02). Value 0 indicates habitat similarity and value 1 indicates habitat dissimilarity.

Mantel tests revealed a positive relationship between microbial Bray-Curtis dissimilarities and habitat dissimilarities in both species, and this effect was strongest in three-spined stickleback (scenario 2, three-spined stickleback: *r* = 0.65, *p*-value = 0.03; nine-spined stickleback: *r* = 0.54, *p*-value = 0.02, Table 3; Figure 8). These results remained significant after accounting for Euclidean distance (three-spined stickleback: *r* = 0.67, *p*-value = 0.01; nine-spined stickleback: *r* = 0.50, *p*-value = 0.03, Table 3). In contrast, there was no significant relationship between Euclidean distance and microbial Bray-Curtis dissimilarities (scenario 1, three-spined stickleback: *r* = 0.08, *p*-value = = 0.26; nine-spined stickleback: *r* = 0.13, *p*-value = 0.19, Table 3; Supplementary Figure S3) or between colonisation history and microbial Bray-Curtis dissimilarities (scenario 3, three-spined stickleback: *r* = −0.29, *p*-value = 0.95; nine-spined stickleback: *r* = −0.22, *p*-value = 0.88, Table 3; Supplementary Figure S4).

**Table 3:** Mantel tests statistics for both host species.

Test	Matrices	Three-spined stickleback	Nine-spined stickleback
Simple Mantel test	X = Habitat; Y = Beta diversity	*R* = 0.65; *p*-value = **0.03**	*R* = 0.54; *p*-value = **0.02**
	X = Geographic distance; Y = Beta diversity	*R* = 0.08; *p*-value = 0.26	*R* = 0.13; *p*-value = 0.19
	X = Colonisation history; Y = Beta diversity	*R* = −0.29; *p*-value = 0.95	*R* = −0.22; *p*-value = 0.88
Partial Mantel test	X = Habitat; Y = Beta diversity; Z = Geographic distance	*R* = 0.67; *p*-value = **0.01**	*R* = 0.50; *p*-value = **0.03**
	X = Colonisation history; Y = Beta diversity; Z = Geographic distance	*R* = −0.36; *p*-value = 0.96	*R* = −0.35; *p*-value = 0.92
	X = Geographic distance; Y = Beta diversity; Z = Habitat	*R* = −0.11; *p*-value = 0.71	*R* = 0.005; *p*-value = 0.44
	X = Geographic distance; Y = Beta diversity; Z = Colonisation history	*R* = 0.19; *p*-value = 0.12	*R* = 0.23; *p*-value = 0.09

*R* denotes the Mantel test statistic. Beta diversity denotes Bray-Curtis distance based matrix generated from the ASV table. Significant results are shown in bold.

## 4. Discussion

Here, we characterised the gut microbiota of two co-existing and phylogenetically related stickleback species using bacterial 16S rRNA (V3-V4) gene sequencing. To understand how host habitat and host factors shape the sticklebacks’ microbiota, we investigated the diversity of the gut microbiota in the two species across populations from freshwater and brackish water habitats. First, microbial communities were clustered by location and habitat, rather than by species, and there was a substantial similarity between the microbial communities of the two host species from the same locations. Second, α diversity was on average higher in nine-spined stickleback, while habitat was a stronger determinant of β diversity in three-spined stickleback.

### 4.1. Microbial diversity shared between host species

The most dominant phyla found in our study populations included Proteobacteria, Actinobacteriota, Firmicutes, Planctomycetota, Chloroflexi and Cyanobacteria. Overall, the similarity between the three-spined and nine-spined stickleback gut microbiota at phylum level was strong. This comes as no surprise, since numerous studies, both on wild as well as lab-reared populations, detected the same dominant phyla in different fish species (Baldo et al., 2015; Estruch et al., 2015; Fietz et al., 2018; Kim et al., 2021; Abdelhafiz et al., 2022; Xu et al., 2022). The single phylum that dominates the gut microbiota of most fishes is the Proteobacteria (Roeselers et al., 2011; Sullam et al., 2015). The presence of dominant phyla is thus conserved across many fish species, but their abundance is affected by different environmental and host-related factors (Kim et al., 2021). The bacterial phyla observed in stickleback guts are phyla that help in homeostasis and nutrient uptake. This includes Proteobacteria, which aid in digestion of complex sugars (Colston and Jackson, 2016) and Actinobacteriota, which help inhibit pathogens and lactic acid fermentation (Colston and Jackson, 2016). Both stickleback species also hosted Cyanobacteria in every single location. The presence of Cyanobacteria suggests that they are important food sources (Xu et al., 2022). Similar observations were made in Asian silver carp (*Hypophthalmichthys molitrix*) and gizzard shad (*Dorosoma cepedianum*) by Ye et al. (2014), who attribute the presence of Cyanobacteria to their role as the fish’s primary food source.

At the genus level, *Rickettsiella, Clostridium sensu stricto 1*, *Aurantimicrobium, Candidatus bacilloplasm* and *PeM15* dominated the microbiome of both stickleback species. The genus *Clostridium* is widely distributed in the animal intestinal community, and many *Clostridium* species may function as mutualistic symbionts with their hosts (Lopetuso et al., 2013). *Clostridium sensu stricto 1* is found in both species and has the ability to digest proteins. Thus, certain bacteria that produce proteases (like *C. sensu stricto 1*) may aid three-spined and nine-spined sticklebacks in using nutrients and obtaining energy from diets high in protein (e.g., aquatic insects and zooplankton) (Schwab et al., 2011). In the three-spined stickleback, a positive relationship has been reported between the abundance of *Clostridiaceae* taxa and the expression of immune genes (Fuess et al., 2021).

Few studies have tested or reported how much overlap there is between populations of coexisting species at the same locations. We found that 7% to 21% ASVs were shared between the populations of the two host species at the same locations. Likewise, in lake whitefish (*Coregonus clupeaformis*), between 22% and 65% (mean ~ 44%) of genera were shared between sympatric species within lakes (Sevellec et al., 2018). However, this study only considered the core ASVs to calculate the shared fraction of the microbiome, while here the total number of ASVs were taken into account. Other studies have reported the overlap among ecotypes within species (Sullam et al., 2015) or between conspecifics from multiple locations (Baldo et al., 2019).

### 4.2. Microbial diversity unique to each host species

Despite the strong similarities between three-spined and nine-spined stickleback populations from the same locations, alpha diversity was overall higher in nine-spined stickleback (Figure 5). This was also confirmed by the higher number of ASVs in the nine-spined stickleback (Figure 2). Higher microbial diversity suggests broader niche use in the nine-spined stickleback, which is consistent with the observation that nine-spined stickleback occupies a slightly higher trophic position (Thorburn et al., 2022). A comparable study by Fietz et al. (2018) observed a similar alpha diversity pattern in sand lance fishes (*Ammodytes tobianus* and *Hyperoplus lanceolatus*) from the Baltic Sea, with higher alpha diversity in *A. tobianus*. In case of sympatric salmonids, the pattern of microbial diversity was similar in brackish water and freshwater habitats, with lake whitefish (*Coregonus clupeaformis*) showing higher alpha diversity than Arctic char (*Salvelinus alpinus*) (Element et al., 2020). The authors reported that it is possible that the diet of lake whitefish is more diverse than that of Arctic char, which in turn may influence microbial richness and diversity (Element et al., 2020). Interestingly, within a population of three-spined stickleback and Eurasian perch (*Perca fluviatilis*), an opposite pattern was observed at the individual level, as individuals with a high diet diversity had low microbial diversity and vice versa (Bolnick et al., 2014b). This result was confirmed with experimental diet manipulations in the three-spined stickleback, where a much lower variation in intestinal microbiota was observed in a mixed diet treatment than in a simple diet treatment (Bolnick et al., 2014b). Finally, Xu et al. (2022) reported highest alpha diversity in a herbivore fish, followed by a carnivore, and then a omnivore fish. Microbial alpha diversity is probably connected with diet (Bolnick et al., 2014b,c; Baldo et al., 2015; Element et al., 2020; Xu et al., 2022). Stomach content analyses may help us to better understand how local environmental conditions affect alpha diversity of the gut microbiome.

In both stickleback species, the alpha diversity of the gut microbial communities varied substantially between populations. Across all locations, Simpson diversity in nine-spined stickleback correlated with Simpson diversity in three-spined stickleback. However, alpha diversity did not correlate with salinity or distance to the coast in either species. Few studies have investigated the determinants of fish gut microbiota alpha diversity across populations and species, which includes habitat, pollution, and diet (Bolnick et al., 2014b; Solovyev et al., 2019; Degregori et al., 2021; Kim et al., 2021). For instance, the gut microbiota of Atlantic salmon (*Salmo salar*) kept in sea cages was more diverse than the gut microbiota of salmon in freshwater (Wang et al., 2021; Morales-Rivera et al., 2022). In two sand lance species of the Baltic Sea, it has been observed that brackish populations for *A. tobianus* had higher Shannon and Chao1 indices than marine populations, but no such difference was observed in *H. lanceolatus* (Fietz et al., 2018). In an experimental setting, Fuess et al. (2021) showed a positive association between microbial alpha diversity and the expression of host immune genes in the three-spined stickleback. Yet, it remains unclear to what extent immunological or any other biological responses affect alpha diversity in natural populations and how this might differ between species.

We observed a strong habitat effect on beta diversity in the two species, since the composition of the microbiome of freshwater populations differed consistently from the composition of the microbiome in brackish water populations. Mantel tests indicated that habitat divergence rather than colonisation history correlated with beta diversity, and this effect remained significant even after correction for geographic distance. A meta-analysis across fish species and populations confirmed that freshwater and marine fish often differ in their gut microbiota communities (Sullam et al., 2012; Kim et al., 2021). The composition of microbiota communities are often shaped by environmental factors, and are also to some extent reflective of their environmental microbial communities (Smith et al., 2015; Dulski et al., 2020). In our study, the fact that the microbial communities of the two host species at a given location are rather similar underlines the importance of the environment in shaping the fish gut microbiota.

Our analysis of beta diversity based on both weighted UniFrac and Bray-Curtis matrices revealed that habitat and location explained a somewhat larger proportion of variation in gut microbiota communities in three-spined stickleback than in nine-spined stickleback. This result was also confirmed with partial Mantel tests with a stronger correlation between Bray-Curtis matrices and habitat dissimilarities. One potential explanation for this stronger effect of habitat divergence is the level of adaptive divergence among host populations. Adaptive divergence among populations and ecotypes of three-spined stickleback is common (McKinnon and Rundle, 2002; Raeymaekers et al., 2007; Hendry et al., 2009; Feulner et al., 2013; Guo et al., 2015), and in our study area, the level of adaptive divergence is markedly stronger in three-spined stickleback than in nine-spined stickleback (Raeymaekers et al., 2017; Bal et al., 2021). So, it could be that the populations of three-spined stickleback in our study area have experienced stronger selection pressures than the populations of nine-spined stickleback, and that this selection history has also led to stronger divergence at the microbiome level. Yet, the weaker effect of habitat divergence in nine-spined stickleback than in three-spined stickleback is not in line with our expectation that microbiome-mediated plasticity could facilitate the freshwater-brackish water transition in this species. Further studies are needed to better understand to what extent the microbiome can play a role in habitat transition.

## 5. Conclusion

Local environmental conditions were a major determinant of the composition of the microbial communities in both host species. Since we did not detect any effect of historical colonisation, we conclude that habitat use is the strongest determinant of microbial diversity. The effect of the local environment was especially pronounced in the three-spined stickleback, which might mirror its stronger propensity for local adaptation. These findings contribute to our understanding of the determinants of host-associated microbial diversity in nature, which will help us to further understand the larger evolutionary patterns that occur between hosts and their associated microbiota.

## Data availability statement

The data presented in the study are deposited in the Sequence Read Archive (SRA) repository, accession number PRJNA942318.

## Ethics statement

The animal study was approved by Ethical Commission Animal Experiments of KU Leuven Belgium. The study was conducted in accordance with the local legislation and institutional requirements.

## Author contributions

AS, JR, JF, and VK: conceptualization. JR: funding acquisition and supervision. AS, TB, and JR: fieldwork. JR and VK: chemicals and resources. AS, MK, and PS: molecular work. AS, CE, and JR: formal analysis. AS and JR: project administration and writing-original draft. AS, CE, JF, VK, TB, MK, PS, YA, and JR: review & editing. All authors contributed to the article and approved the submitted version.
