# Supplementary material for: Host habitat rather than evolutionary history explains gut microbiome diversity in sympatric stickleback species
Source: Front Microbiol. 2023 Oct 12;14:1232358. doi: 10.3389/fmicb.2023.1232358 (PMC10601471; doi:10.3389/fmicb.2023.1232358)
Supplement: Supplementary file 1 [file Data_Sheet_1.pdf]

Supplementary Material:

Host habitat rather than evolutionary history explains gut microbiome diversity in sympatric stickleback species

Authors:
Aruna M. Shankregowda* (1)
Prabhugouda Siriyappagouder (1)
Marijn Kuizenga (1)
Thijs M. P. Bal (1)
Yousri Abdelhafiz (1)
Christophe Eizaguirre (2)
Jorge M. O. Fernandes (1)
Viswanath Kiron (1)
Joost A. M. Raeymaekers (1)

* Corresponding author

Author information:

1) Faculty of Biosciences and Aquaculture, Nord University, Bodø, Norway
2) School of Biological and Behavioural Sciences, Queen Mary University of London, London, UK

Supplementary methods:

A total of 5,987,681 high-quality reads were obtained from 253 samples (due to bad quality, three samples were removed after library preparation and bioinformatic analysis). The average number of reads per sample was 23,666 (**Supplementary Table S1**). Reads were rarefied at 9000 to consider variation in read count in different samples, which resulted in 39 samples being removed (26 samples of three-spined stickleback and 13 samples of nine-spined stickleback).

Supplementary Tables:

Table S1: Sample collection details by location and species, including the number of reads after quality filtering, but before rarifying. N denotes sample size (number of individuals) after quality filtering.

Species	Habitat	River Basin/Region	location	Coordinates (lat; long)	N	Raw reads	Filtered reads	Merged reads	Non-chimeric reads
Three-spined stickleback	Brackish water	Coastal lowland	LO1	51.35296; 3.43384	16	384845	314676	231622	210880
			LO6	51.2828; 3.58352	15	607039	493153	413641	386944
	Fresh-water	West Scheldt	L14	51.05868; 3.41307	16	814393	670185	637884	626031
			LOK	51.09531; 3.99193	16	385628	311498	238160	216607
		East Scheldt	DIEST	50.9729; 5.0453	16	467218	377799	320771	299774
			TON	50.796217; 5.419395	15	601257	486372	362764	342834
		Meuse	ELS	51.27573; 5.34615	16	449589	367224	268267	251808
			NET	51.08464; 5.69278	16	751789	622712	575077	562687
Nine-spined stickleback	Brackish water	Coastal lowland	LO1	51.35296; 3.43384	16	581724	477027	391633	370076
			LO6	51.2828; 3.58352	15	659841	542500	375506	338984
	Fresh-water	West Scheldt	L14	51.05868; 3.41307	16	687640	573351	534612	526571
			LOK	51.09531; 3.99193	16	599376	491047	399966	378608
		East Scheldt	DIEST	50.9729; 5.0453	16	613157	496961	391658	365990
			TON	50.796217; 5.419395	16	608369	495838	380733	355632
		Meuse	ELS	51.27573; 5.34615	16	481586	384840	269345	247195
			NET	51.08464; 5.69278	16	681176	560125	515644	507060

Table S2: Two-way ANOVA for the effect of location, host species and the interaction between location × host species on the abundance of selected phyla.

Phylum		Location	Host species	Location : Host species
Proteobacteria	Df	7	1	7
	F	1.66	0.83	3.65
	Sum Sq	53568	3846	117643
	P-value	0.11	0.36	**0.0009**
Actinobacteriota	Df	7	1	7
	F	2.23	6.45	1.71
	Sum Sq	5303	2186	4064
	P-value	**0.03**	**0.01**	0.10
Planctomycetota	Df	7	1	7
	F	4.52	4.07	6.38
	Sum Sq	85456	11006	120671
	P-value	**0.0001**	**0.04**	**<0.0001**
Firmicutes	Df	7	1	7
	F	4.79	1.06	1.61
	Sum Sq	9366	297	3148
	P-value	**<0.0001**	0.30	0.13
Chloroflexi	Df	7	1	7
	F	9.61	8.28	1.25
	Sum Sq	28342	3488	3689
	P-value	**<0.0001**	**0.004**	0.27

Df denotes degrees of freedom, F denotes F statistic and Sum Sq denotes the variation attributed to the error. Significant results are shown in bold.

Table S3: Two-way ANOVA for the effect of location, host-species and the interaction between location × host species on the abundance of selected genera.

Genera		Location	Host species	Location : Host species
Rickettsiella	Df	7	1	7
	F	9.19	4.92	3.58
	Sum Sq	1038	79.4	405.2
	P-value	**<0.0001**	**0.02**	**0.001**
Aurantimicrobium	Df	7	1	7
	F	5.56	4.45	0.99
	Sum Sq	312	35	55
	P-value	**<0.0001**	**0.03**	0.43
Candidatus_Bacilloplasma	Df	7	1	7
	F	5.93	0.0001	5.48
	Sum Sq	86.39	0.00	79.77
	P-value	**<0.0001**	0.99	**<0.0001**
PeM15	Df	7	1	7
	F	4.8	19.75	2.57
	Sum Sq	195.93	114.55	104.51
	P-value	**<0.0001**	**<0.0001**	**0.01**
Clostridium_sensu_stricto_1	Df	7	1	7
	F	2.62	1.84	2.31
	Sum Sq	83.8	8.42	73.81
	P-value	**0.01**	0.17	**0.02**

Df denotes degrees of freedom, F denotes F statistic and Sum Sq denotes the variation attributed to the error. Significant results are shown in bold.

Supplementary Figures:

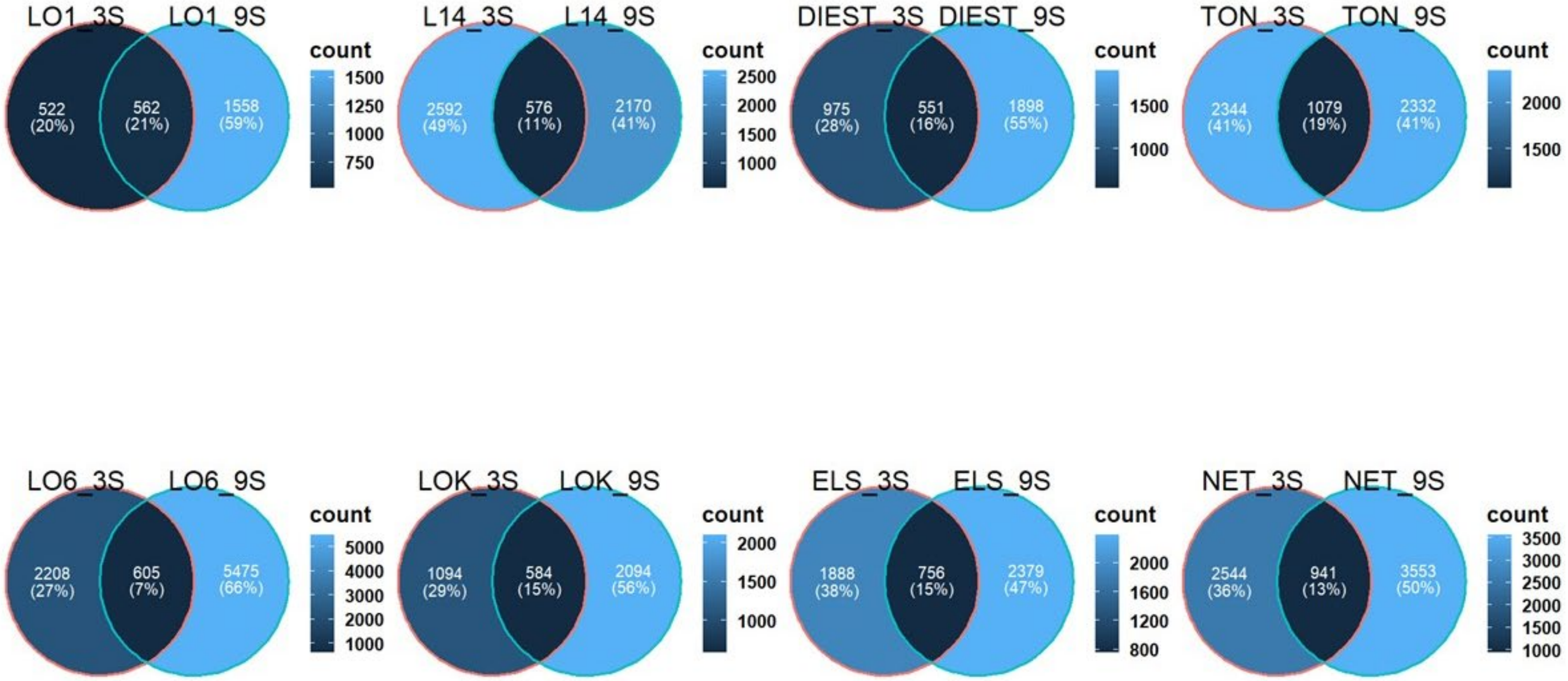

Figure. S1 Shared and distinct ASVs of the three-spined (3S) and nine-spined (9S) stickleback populations from eight locations. The colour scale indicates ASVs number.

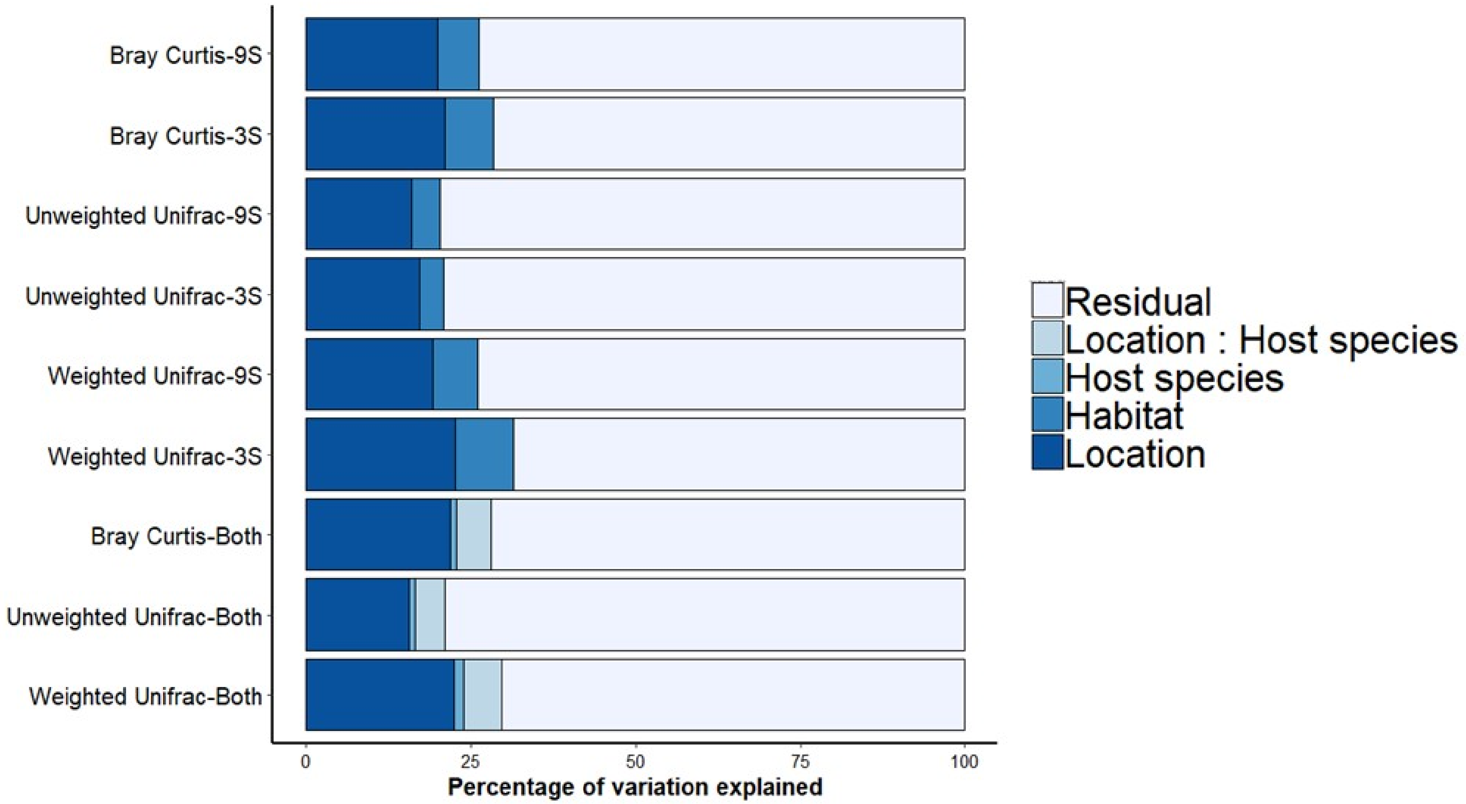

Figure. S2 The proportion of variance in microbial communities in both host species explained by habitat and location, for three different diversity measures.

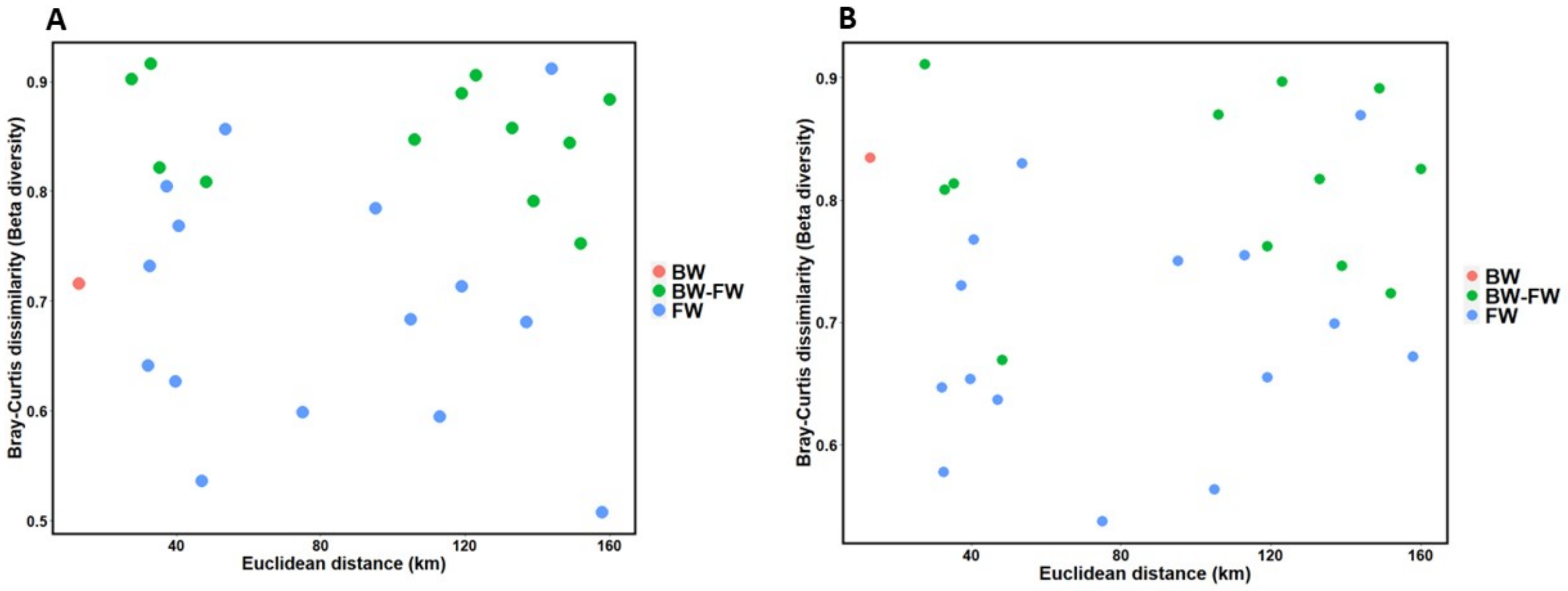

Figure. S3 Mantel test for isolation by distance between the matrix of Euclidean distances and Bray Curtis dissimilarities. **(A)** three-spined stickleback (R = 0.08, P-value = 0.26), **(B)** nine-spined stickleback (R = 0.13, P-value = 0.19). Red dots mark brackish water (BW) population pairs, blue dots mark freshwater (FW) population pairs, and green dots mark brackish water - freshwater (BW-FW) population pairs.

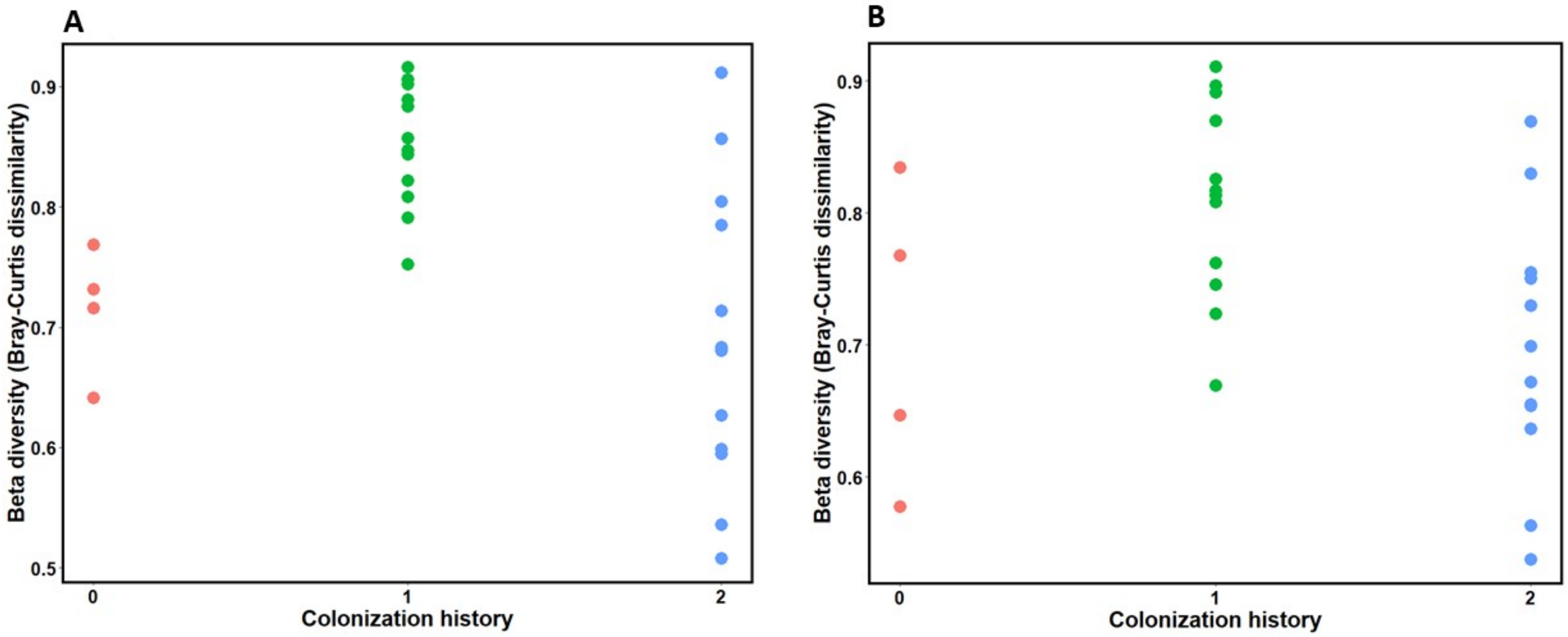

Figure. S4 Mantel test for isolation by distance between the matrix of colonization history and Bray Curtis dissimilarities. **(A)** three-spined stickleback (R = -0.29, P-value = 0.95), **(B)** nine-spined stickleback (R = -0.22, P-value = 0.88). Red dots mark brackish water population pairs (no freshwater colonisation history) and freshwater population pairs from the same watershed (same colonisation history), green dots mark brackish water-freshwater pairs (direct ancestry) and blue dots mark freshwater populations from different watersheds (independent colonisation).
